# Intracellular Antioxidant Activity of Grape Skin Polyphenolic Extracts in Rat Superficial Colonocytes: *In situ* Detection by Confocal Fluorescence Microscopy

**DOI:** 10.3389/fphys.2016.00177

**Published:** 2016-05-27

**Authors:** M. Elena Giordano, Ilaria Ingrosso, Trifone Schettino, Roberto Caricato, Giovanna Giovinazzo, M. Giulia Lionetto

**Affiliations:** ^1^Department of Biological and Environmental Sciences and Technologies, University of SalentoLecce, Italy; ^2^Institute of Science of Food Production, Unit of Lecce, National Research Council of ItalyLecce, Italy

**Keywords:** colon, antioxidant, polyphenols, hydrogen peroxide, H2DCFDA

## Abstract

Colon is exposed to a number of prooxidant conditions and several colon diseases are associated with increased levels of reactive species. Polyphenols are the most abundant antioxidants in the diet, but to date no information is available about their absorption and potential intracellular antioxidant activity on colon epithelial cells. The work was addressed to study the intracellular antioxidant activity of red grape polyphenolic extracts on rat colon epithelium experimentally exposed to prooxidant conditions. The experimental model chosen was represented by freshly isolated colon explants, which closely resemble the functional, and morphological characteristics of the epithelium *in vivo*. The study was carried out by *in situ* confocal microscopy observation on CM-H2DCFDA charged explants exposed to H_2_O_2_ (5, 10, and 15 min). The qualitative and quantitative polyphenolic composition of the extracts as well as their *in vitro* oxygen radical absorbing capacity (ORAC) was determined. The incubation of the explants with the polyphenolic extracts for 1 h produced a significant decrease of the H_2_O_2_ induced fluorescence. This effect was more pronounced following 15 min H_2_O_2_ exposure with respect to 5 min and it was also more evident for extracts obtained from mature grapes, which showed an increased ORAC value and qualitative peculiarities in the polyphenolic composition. The results demonstrated the ability of red grape polyphenols to cross the plasma membrane and exert a direct intracellular antioxidant activity in surface colonocytes, inducing a protection against pro-oxidant conditions. The changes in the polyphenol composition due to ripening process was reflected in a more effective antioxidant protection.

## Introduction

Oxidative stress condition is defined as a disturbance in the balance between antioxidant defenses and the production of reactive oxygen species (ROS), such as superoxide anions (${\text{O}}_{2}^{-}$), hydrogen peroxide (H_2_O_2_), hydroxyl radical (−OH), as well as reactive nitrogen species (RNS) such as nitric oxide (NO) and peroxynitrite (OONO−). Oxidative stress facilitates the initiation of radical chain reactions known to augment lipid peroxidation, DNA oxidation and protein damage in cells with detrimental effects on the organism functions.

The gastrointestinal tract is particularly exposed to reactive species via exogenous and endogenous routes. A remarkable exogenous source is represented by the diet, such as pollutant contaminated food intake or oxidant compounds present in foods. For example consumption of a meal containing oxidized and oxidizable lipids gives rise to a postprandial oxidative stress in the lumen of the digestive tract because of peroxidation of lipids present in the meal during digestion (Ursini and Sevanian, 2002). Examples of endogenous route are represented by the nitrosation of precursors of nitrosamines and increased production of reactive oxygen and nitrogen species by *Helicobacter pylori infection* at the gastric level (Handa et al., 2011), or increased production of H_2_O_2_ by numerous stimulated phagocytes and from some bacteria species in chronic gastrointestinal inflammatory conditions (Nathan, 2002; Strus et al., 2009).

A number of diseases of the gastrointestinal tract are associated with the imbalance in the cellular redox system and are related to increased levels of reactive species (Kim et al., 2012; Bhattacharyya et al., 2014). Although the small amounts of ROS produced under normal conditions have a protective effect, such as modulation of the immune-mediated attack against extrinsic pathogens and activation of protective signaling pathways against inflammation, ROS as well as RNS overproduction under abnormal conditions can contribute to the immediate development of inflammatory processes in the gastrointestinal tract (Kim et al., 2012). These superficially opposite functions can explain why ROS play key roles in the pathogenesis of numerous chronic inflammatory disorders of the GI tract, including reflux esophagitis, Barrett's esophagus, *H. pylori* induced gastritis, and inflammatory bowel diseases such as ulcerative colitis and Crohn's disease (Kim et al., 2012). In addition to their critical implications in inflammatory processes at the gastrointestinal level, ROS are directly and indirectly involved in the multi-stage process of carcinogenesis (Klaunig et al., 2011).

Several epidemiological studies have indicated that regular intake of vegetables, fruit, and beverages, such as red wine, is associated with a decreased incidence of cancer and coronary diseases. The protective effect has been attributable, at least in part, to polyphenols. It is known that polyphenols may have health-protective effects on the gastrointestinal tract preserving antioxidant defenses and complexing transition metals (Frei and Higdon, 2003). Grape products are known to contain high levels of polyphenols, which are predominantly found in skins and seeds. Therefore, grape derivatives have been attracting growing attention thanks to their high polyphenol content, which may have potential gut health benefits (Forester and Waterhouse, 2009; Lionetto et al., 2011; Giovinazzo and Grieco, 2015). Wine consumption has been reported to have an inverse association with colorectal cancers (Anderson et al., 2005), and polyphenols in wine have been suggested to be responsible for the effects observed in these epidemiological studies. Red wine polyphenols have proven capable to inhibit peroxidation reactions at the gastric level by shifting the balance of reactions by pro-oxidants to antioxidant (Krul et al., 2004). Polyphenols are able to react in one-electron reactions with free radicals *in vitro*. Recently, Forman et al. (2014) argued against their *in vivo* scavenging of radicals, but ascribed their physiological mechanism of antioxidant action to the paradoxical oxidative activation of Nrf2(NF-E2) related factor 2 signaling pathway that maintains protective oxidoreductase and their nucleophilic substrate.

Although there is some controversy regarding bioavailability of polyphenols (Manach et al., 2004; McGhie and Walton, 2007), it is known that in the gastrointestinal tract lumen these substances can reach concentrations up to several hundred micromolar, in particular at the colon level (Romier et al., 2009). It is known that most of the ingested polyphenols are not absorbed in the small intestine, but pass to the colon where they are partly degraded by the action of the microbiota, to give rise to a plethora of small phenolic acid and aromatic catabolites that are absorbed into the circulatory system (Romier et al., 2009). To date no information is available about the absorption and the potential antioxidant intracellular activity of polyphenols on colon surface cells.

The aim of the present work was to study the intracellular antioxidant activity of red grape polyphenolic extracts on rat colon epithelium experimentally exposed to prooxidant conditions. Freshly isolated explants were used as experimental model because they closely resemble the functional and morphological characteristics of the epithelium *in vivo* (Bjorkman et al., 1986). Confocal microscopy explants charged with the cell-permeant fluorescent redox indicator probe CM-H2DCFDA was applied. The intracellular antioxidant activity was assessed *in situ* as inhibition of the probe florescence experimentally induced by H_2_O_2_ exposure. The obtained values were compared with the intracellular antioxidant activity of the membrane permeable antioxidant Trolox, a synthetic analog of vitamin E, which acts as free radical scavengers. It is known that the polyphenolic composition of red grape skin changes during the ripening process (Zamboni et al., 2010). Therefore, in order to assess if changes in the polyphenolic composition can influence the potential intracellular antioxidant activity of the extracts, two red grape polyphenolic extracts were utilized: one obtained from grapes sampled 1 month before the complete maturation, and the second obtained from fully ripe grapes. Total phenolic content and oxygen radical absorbing capacity (ORAC) of the extracts were analyzed and compared with the effects observed on superficial colonocytes in terms of intracellular antioxidant activity.

## Materials and methods

### Materials

Male Wistar rats (70–100 g) were obtained from Harlan (Carezzana, Italy) and housed individually in animal cages at a temperature of 22 ± 1°C with a 12:12 h light-dark cycle and 30–40% humidity. The animals had *ad libitum* access to food and water. This study was carried out in strict accordance with the European Committee Council 106 Directive (86/609/EEC) and with the Italian animal welfare legislation (art 4 and 5 of D.L. 116/92).

Samples of red grape variety (*Vitis vinifera* L.) Primitivo cultivated on South of Italy vineyard (Apulilan region) were obtained from Cantina Due Palme Cellino S. Marco, (BR). The sampling was carried out by taking into account the variability of the positions of the fruit on the cluster, of the cluster on the vine, and of the vine in the vineyard as well as from variations in sun exposure to asses a representative sample of the vineyard. Special care was taken to obtain a good distribution between berries from the inside and the outside of the cluster: one was taken from the top, one from the bottom, and one from the middle of the cluster. Healthy grape berries were snipped from the clusters, the skin from 50 healthy berries, randomly collected, were manually separated from pulp and seeds, weighed then frozen in liquid nitrogen, lyophilized and stored at 5°C until analysis. The representative samples were harvested at two ripening stage (in August and September 2011 and 2012).

All chemicals were reagent grade. The fluorescent dye CM-H2DCFDA was purchased from Life Technologies–Molecular Probes. *Trans*-resveratrol and *trans*-piceid were obtained from ICN Biomedicals (South Chillicothe Road, Aurora, Ohio), quercetin3β-D glucoside, myricetin and kaempferol from ExtraSynthese (Genay, France), quercetin, caffeic acid, p-coumaric acid and caftaric acid from Sigma (St. Louis, MO, USA) as well as all other reagents when not otherwise specified.

### Intracellular antioxidant activity assessment of superficial colonocytes by confocal microscopy

#### Isolation of colon explants and loading with CM-H2DCFDA

Rats were sacrificed by cardiac arrest through the use of diethyl ether and the distal colon was removed by aseptic technique. The isolated colon was washed with sterile Hepes-Tris buffer solution containing (in mM): NaCl 140, KCl 5, MgCl_2_ 1, CaCl_2_ 2, Hepes 10, glucose 10 adjusted to pH 7.4 with Tris or Hepes. Then, the colon was opened longitudinally with surgical scissors and cut into explants of about 0.6 cm^2^ in size. Individual explants were washed with sterile Hepes-Tris buffer solution and placed in 60-mm plastic tissue-culture dishes with the epithelium facing the gas-medium interface. Cell viability was assessed by the Trypan blue exclusion test. The viability of the colon superficial colonocytes in the explants were determined by their ability to exclude the vital stain, trypan blue as assessed by bright-field microscopy on small tissue fragments (about 0.06 cm2) dispensed on a poly-L-lysine coated slides.

Small fragments of freshly isolated colon explants were immediately incubated in Tris-buffer solution (see above) with the cell-permeant fluorescent probe CM-H2DCFDA (5-(and-6)-chloromethyl-2′,7′-dichlorodihydrofluorescein diacetate, final concentration 5 μM) for 30 min at 37°C.

In the cell this is hydrolysed to the DCFH carboxylate anion, which is retained intracellularly. The two-electron oxidation of DCFH produces the fluorescent probe, dichlorofluorescein (DCF).

The tissue fragments were then washed three times with Tris-buffer solution to remove the probe from the extracellular medium.

#### Confocal visualization

Freshly isolated colon explants were exposed to 400 μM H_2_O_2_ for 5, 10, or 15 min, respectively. This concentration was previously (Antico et al., 2013) demonstrated to evoke oxidative stress induced apoptosis in rat colon explants following 24 h of incubation. In the present work it was utilized for very short periods of time (5 and 15 min) to induce oxidative intracellular condition.

Small fragments of colon explants (0.06 mm^2^) charged with CM-H2DCFDA were freshly mounted on a poly-L-lysine coated slide and then viewed using a 100X NA plan apochromatic objective mounted on a NIKON TE300 inverted microscope coupled to a NIKON C1 confocal laser scanning unit (Nikon, Tokyo, Japan). The fluorescent dye CM-H2DCFDA was excited using the Argon 488-nm laser line. Typically, measurements of fluorescence intensity were performed on a group of about 50–70 colon superficial cells per field and represent the average intensity of all fluorescent cells within the selected region of interest (ROI). Each measurement was performed on at least five fields randomically chosen on each explant. Unlabeled preparations were found to exhibit no fluorescence under the conditions used. Images were acquired and analyzed by EZ-C1 NIKON software for quantification of emitted fluorescence intensity.

In order to assess the intracellular antioxidant activity of red grape skin polyphenolic extracts on superficial colonocytes, explants were pre-incubated for 1 h with the extract (1:100 dilution), and then washed three times with the Hepes-Tris buffer solution before to CM-H2DCFDA charging and H_2_O_2_ exposure. The 1:100 dilution was chosen on the basis of preliminary tests with serial dilutions, since it was able to exert the maximum detectable effect. Then the intracellular antioxidant activity was assessed as percentage inhibition of the H_2_O_2_ induced CM-H2DCFDA fluorescence.

### Polyphenolic extract

#### Preparation of the polyphenolic extract

Grape skins were frozen in liquid nitrogen and grinded with a blender until fine powder. The samples (100 mg of dry weight corresponding to 1 g of fresh weight) were treated with 1 ml of 80% methanol for 24 h in the dark. Cell debris was removed by centrifugation **(**4000 rpm**)** for 5 min, the supernatant evaporated to dryness, under reduced pressure, at 40°C and re-dissolved in 80% ethanol (500 μL). Samples were protected from light during the analysis process.

#### Spectrophotometric analysis

The total amount of polyphenols was measured with an optimized Folin-Ciocalteu method (Rigo et al., 2000). Grape skin polyphenol extract (GSPE) dry powders were solubilized in 70% ethanol and stored in the dark at 5°C until use. The total phenolic content in extracts (diluted 50 times with deionized water) was determined by measuring the absorbance of diluted extracts at 765 nm according to the Folin-Ciocalteau colorimetric method. Results were expressed as microgram gallic acid equivalents per milliliter of grape extract (μg GAEs/ml).

Total anthocyanins pigments in skin extracts and hydroalcoholic solutions were measured at 520 nm after dilution in appropriate amounts of 1 N HCl at pH 1.0 (to be between 0.02 and 1 absorbance unit) and are expressed as μg/L malvidin-3-*O*-glucoside equivalents according to Fournand et al. (2006).

#### HPLC analysis of phenolic compounds

Different compounds present in grape skin extracts were separated by RP-HPLC DAD (Agilent 1100 HPLC apparatus). The separation was performed on C18 column (5 UltraSphere run spherical 80 A pore, 25 mm), with a linear gradient from 20 to 60% acetonitrile, in 40 min (solvent A: 1% H3PO4, solvent B: 100% acetonitrile) with a flow of 1 ml/min at 25°C. The chromatographic analysis performed with UV detectors was based on the comparison of results with retention time of external standards. The metabolite concentrations were obtained by deduction from the calibration curves.

Recovery was determined for the overall assay by adding known amounts of different metabolites to the original concentration of the analyzed samples and obtained values are between 85 and 93%.

#### Antioxidant activity determination of the polyphenol extracts by the ORAC (oxygen radical absorbance capacity) assay

The *in vitro* antioxidant capacity of the grape skin polyphenolic extracts was determined by the ORAC assay as previously described (Bleve et al., 2008). The ORAC method utilized in this work was previously described by Ou et al. (2001) which used fluorescein instead of β-phycoerythrin as fluorescent probe because it is more photostable and does not interact with testing substances. In this paper we utilized a further modification of the ORAC-fluorescein method, introduced by Davalos et al. (2004) for manual handling of the assay. Briefly, the reaction was carried out in 75 mM phosphate buffer (pH 7.4) and the final reaction mixture was 2 ml. The diluted polyphenol extract (20 μl) and fluorescein solution (1200 μl, 120 nM) were placed in the fluorimeter cuvette. The mixture was pre-incubated for 15 min at 37°C. Then, the peroxyl radical generator AAPH solution (600 μl, 40 mM) was rapidly added and the fluorescence was recorded every minute for 80 min. Excitation and emission wave lengths were 535 and 560 nm, respectively. A blank (fluorescein + AAPH) containing phosphate buffer instead of the antioxidant solution and four calibration points containing Trolox (1, 2, 4, and 8 μM final concentration) as antioxidant standard were also carried out in each assay. For each fluorescent decay curve (fluorescence vs. time) the area under curve (AUC) was calculated AS follows:

$$\begin{array}{l} \begin{array}{l} \text{AUC}= \end{array}\frac{\sum_{\text{i}=0}^{\text{n}}{\text{f}}_{i}}{{\text{f}}_{\text{0}}} \end{array}$$

where f_0_ is the initial fluorescence reading at 0 min and f_i_ is the fluorescence reading at time i. For each sample the net AUC was calculated by subtracting the AUC corresponding to the blank.

A standard curve (AUC vs. Trolox concentrations) was drown for each assay and the ORAC values, expressed as micromoles of Trolox equivalents, and calculated by interpolating the AUC sample values on the standard curve.

## Statistical analysis

Data are presented as mean ± S.E.M. For multiple comparisons, analysis was by one-way ANOVA or two way ANOVA followed by the Bonferroni *post-hoc* test, as specified in the captions to figures. The GraphPad Prism (version 5) software was used for all the analyses and graphing.

## Results

### Confocal visualization of rat colon surface colonocytes exposed to H_2_O_2_

**Figure 1:**
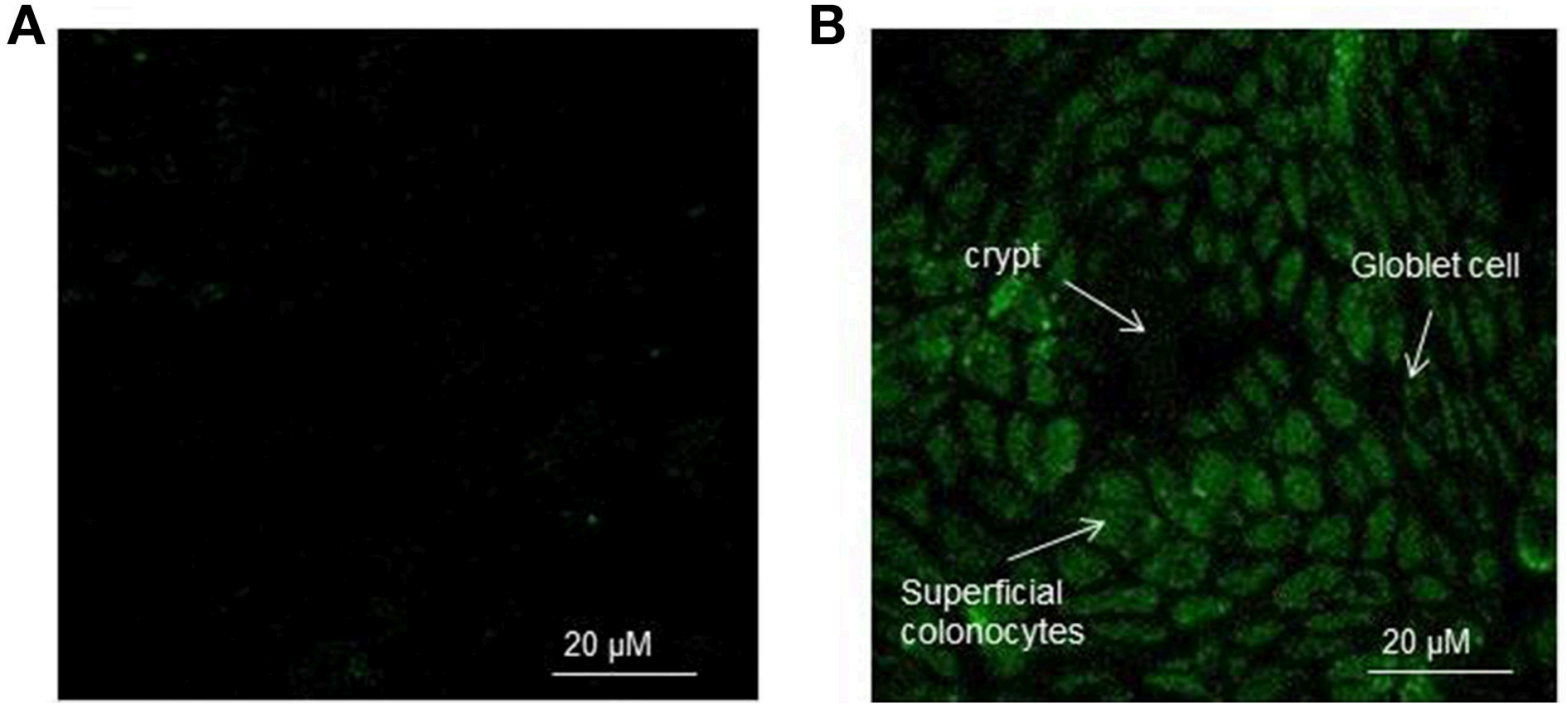
**(A,B)** Representative confocal image of the rat colon surface of control **(A)** and H_2_O_2_ exposed **(B)** explants charged with the CM-H2DCFDA probe. In **(B)** the explants were exposed to 400 μM H_2_O_2_ for 15 min. The epithelial surface was visualized using confocal laser scanning microscopy (see Materials and Methods) at 100X objective.

The appearance of rat colon surface of control and H_2_O_2_ exposed explants charged with the CM-H2DCFDA probe is reported in Figures 1A,B as observed by confocal microscopy. H_2_O_2_ is a major component of ROS and is used extensively as an inducer in oxidative stress models. In control explants the fluorescence signal was very slight and no clear cellular appearance was evident. On the other hand, when the explants were exposed to 400 μM H_2_O_2_ for 15 min, an increase of the intracellular fluorescence of superficial colonocytes was observed. This indicated an increase of the concentration of DCF which is the fluorescent oxidation product of H2DCFDA, widely used as a general marker of cellular oxidation by ROS. It is known the presence of different cell types in the colon surface (Colony, 1996) with absorptive cells as the predominant epithelial cell type. Absorptive cells showed a green fluorescence homogenously distributed in the cell cytoplasm with some more fluorescent spots, particularly following 15 min H_2_O_2_ exposure. This makes the absorptive cells clearly visible in H_2_O_2_ treated explants, with the crypt architecture evident and the superficial colonocytes arranged around a central lumen. On the other hand goblet cells, which secrete mucins, the major components of mucus, appeared as black walls in the epithelium surface (Figure 1B).

**Figure 2:**
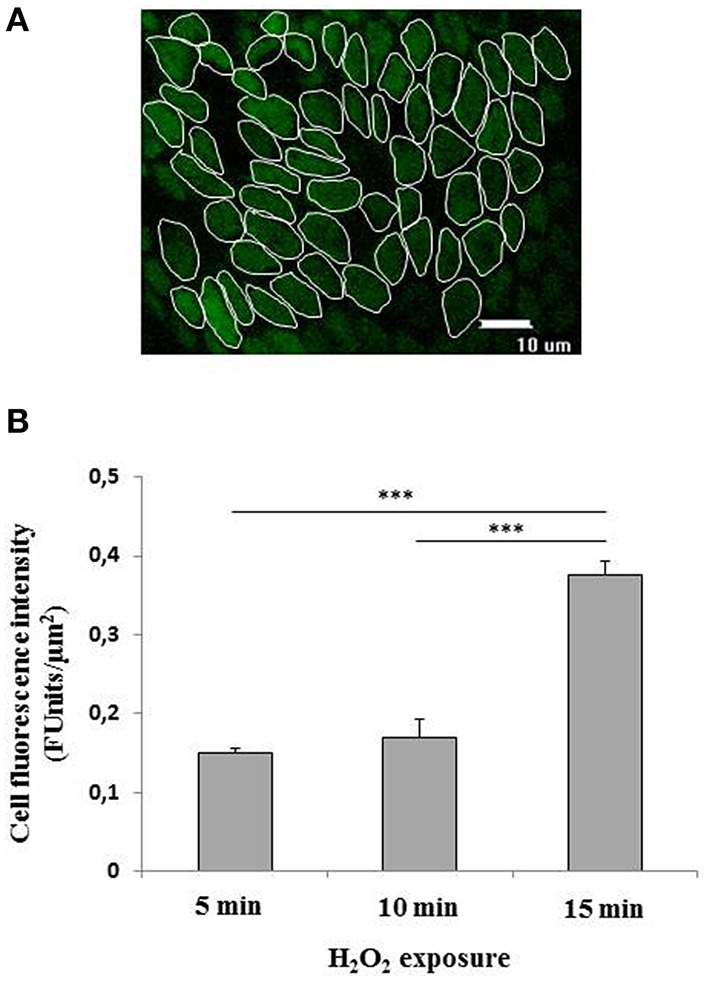
**(A,B)** Quantification of the fluorescence emitted by CM-H2DCFDA charged superficial colonocytes exposed to H_2_O_2_. **(A)** Fluorescence intensity was measured in a group of about 50–70 colon superficial cells per field within selected regions of interest (ROI). At least five fields were randomically chosen on each explant. Three explants were observed for each condition. The epithelial surface was visualized using confocal laser scanning microscopy (see Materials and Methods) at 100X objective. Unlabeled preparations were found to exhibit no fluorescence under the conditions used. **(B)** Fluorescence emitted by CM-H2DCFDA charged superficial colonocytes exposed to 400 μM H_2_O_2_ for 5, 10, and 15 min, respectively. Data are expressed as mean ± S.E.M. The statistical significance of data was assessed by one way ANOVA followed by Bonferroni *post-hoc* test. ^***^P < 0001.

Cell to cell variability in H_2_O_2_ induced fluorescence was assessed by measuring fluorescence intensity per μm^2^ induced by H_2_O_2_ exposure in 50–70 individual cells chosen at random in the ROI (ROI) within the epithelial surface (Figure 2A). Five ROIs were chosen at random in each explant. The fluorescence emitted following the exposure to 400 μM H_2_O_2_ was time-dependent as assessed at 5, 10, and 15 min exposure. In particular, the fluorescence was evident already at 5 min exposure, remained constant in the following 5 min and showed a significant (*p* < 0.001) increase at 15 min exposure with respect to 5 or 10 min (Figure 2B). The background fluorescence of control cells was subtracted from all conditions.

**Figure 3:**
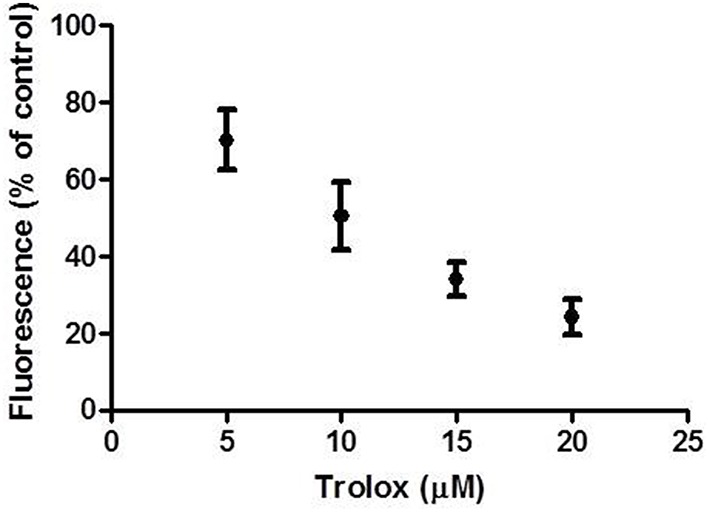
**Effect of Trolox incubation on H_2_O_2_ induced fluorescence**. The graph shows the residual fluorescence (percentage vs. control) recorded from superficial colonocytes of explants pre-incubated with increasing Trolox concentrations for 1 h, charged with CM-H2DCFDA and then treated with 400 μM H_2_O_2_ for 15 min. Control cells were from explants not pre-incubated with Trolox. Data are reported as the mean ± SEM of three replicates.

In order to assess the ability of the experimental set up to detect intracellular antioxidant activity by intracellularly absorbed antioxidant compounds in superficial colonocytes, we incubated the explants with the membrane-permeable antioxidant Trolox for 1 h. Trolox is a synthetic analog of vitamin E, which has been previously demonstrated to act as scavenger on H_2_O_2_-derived ROS (Distelmaier et al., 2012). Figure 3 shows the intracellular radical scavenging activity of Trolox, measured as percentage residual fluorescence (vs. control) of superficial colonocytes (charged with CM-H2DCFDA and then treated with 400 μM H_2_O_2_ for 15 min), plotted against the concentrations of the antioxidant. The H_2_O_2_ induced fluorescence intensity was significantly decreased by Trolox incubation in a dose-dependent manner (Figure 3). On the other hand, when the explants were incubated for 1 h with Tris buffer solution without any antioxidant (negative control) prior than H_2_O_2_ treatment, no significant changes in the H_2_O_2_ induced fluorescence was observed (data not shown).

### Assessment of intracellular antioxidant activity of grape skin polyphenols in rat colon cells

Then, we verified the suitability of this experimental model for the assessment of the potential absorbability and intracellular antioxidant activity of polyphenolic extracts from red grape skin.

#### Characterization of the polyphenolic content and ORAC levels of the polyphenolic extracts

Two extracts were utilized, corresponding to different ripening state of the berries: the first extract was obtained from grapes sampled 1 month before harvest (GSPE 1), while the second was obtained from fully ripe grapes (GSPE 2).

In Table 1 the characterization of the two polyphenol extracts from grape skin and ORAC (expressed in ORAC units) are reported. Grape skin extracts contained a complex mixture of structurally different bioactive molecules (Giovinazzo and Grieco, 2015). We investigated the antioxidant effects of polyphenols mainly represented in the grape skin extracts GSPE1 and GSPE2, such as flavonoids, namely flavonols (kaempferol, quercetin, quercetin 3β-D glucoside, myricetin), as well as non-flavonoids such as soluble acids (coumaric acid, caftaric acid, gallic acid) stilbenes (*trans*-resveratrol, *trans*-piceid) and anthocyanins. The total phenols amount in the two grape skin extracts was not significantly different (Table 1). On the other hand, differences were detected in the amount of specific polyphenols. For example in GSPE1 extract the amount (11%) of flavonol class was higher than in GSP2 extract (3%). This finding was due to the absence of Quercetin 3b-D glucoside in GSPE2 (grape at the harvesting stage). Stilbenes significantly changed during maturation, indeed, in GSPE 2 *trans*-resveratrol and *trans*-piced concentration was more balanced. The total amount of anthocyanins increased (61 vs. 49%) in mature skin grape extracts (GSPE 2). As reported in Table 1, the ORAC of the extracts, assessed by the ORAC assay, slightly but significantly (*P* < 0.05) increased with the ripening process.

**Table 1:** **Characterization of GSPE1 and GSPE2 different classes of polyphenol, total polyphenol content (Folin Ciocalteu assay), and antioxidant capability (ORAC assay)**.

**Groups**	**Compounds**	**GSPE 1 (μg/mL)**	**%**	**GSPE 2 (μg/mL)**	**%**
Soluble acids	*p-Coumaric acid*	0.45 ± 0.03		0.50 ± 0.2	
	*Caftaric acid*	7.19 ± 1.2^*^		5.45 ± 0.03	
	*Gallic acid*	9.66 ± 0.2^*^		8.27 ± 0.7	
			44.6		36
Flavonols	*Kaempferol*	0.15 ± 0.05		0.13 ± 0.02	
	*Quercetin*	0.91 ± 0.05		0.96 ± 0.06	
	*Quercetin 3 β D Glucoside*	3.13 ± 0.5^*^		n.d.	
	*Myricetin*	0.10 ± 0.01		0.11 ± 0.05	
			11		3
Stilbenes	*trans-Resveratrol*	0.05 ± 0.01		0.30 ± 0.1^*^	
	*lrans-Piceid*	1.28 ± 0.4^*^		0.69 ± 0.1	
			3.4		2.5
Anthocy anins		19.03 ± 1.5		24.32 ± 1.2^*^	
			49		61
Total phenols	(μg GAEs/mL)	38.92 ± 1.5	100	40.1 ± 1.5	100
ORAC/mL		255.6+13.2		293.8+0.8^*^	

*Polyphenol concentration, of both grape skin extracts, is expressed in μg/mL*.

* *P < 0.05*.

#### Intracellular antioxidant activity of the polyphenolic extracts on surface colon cells

**Figure 4:**
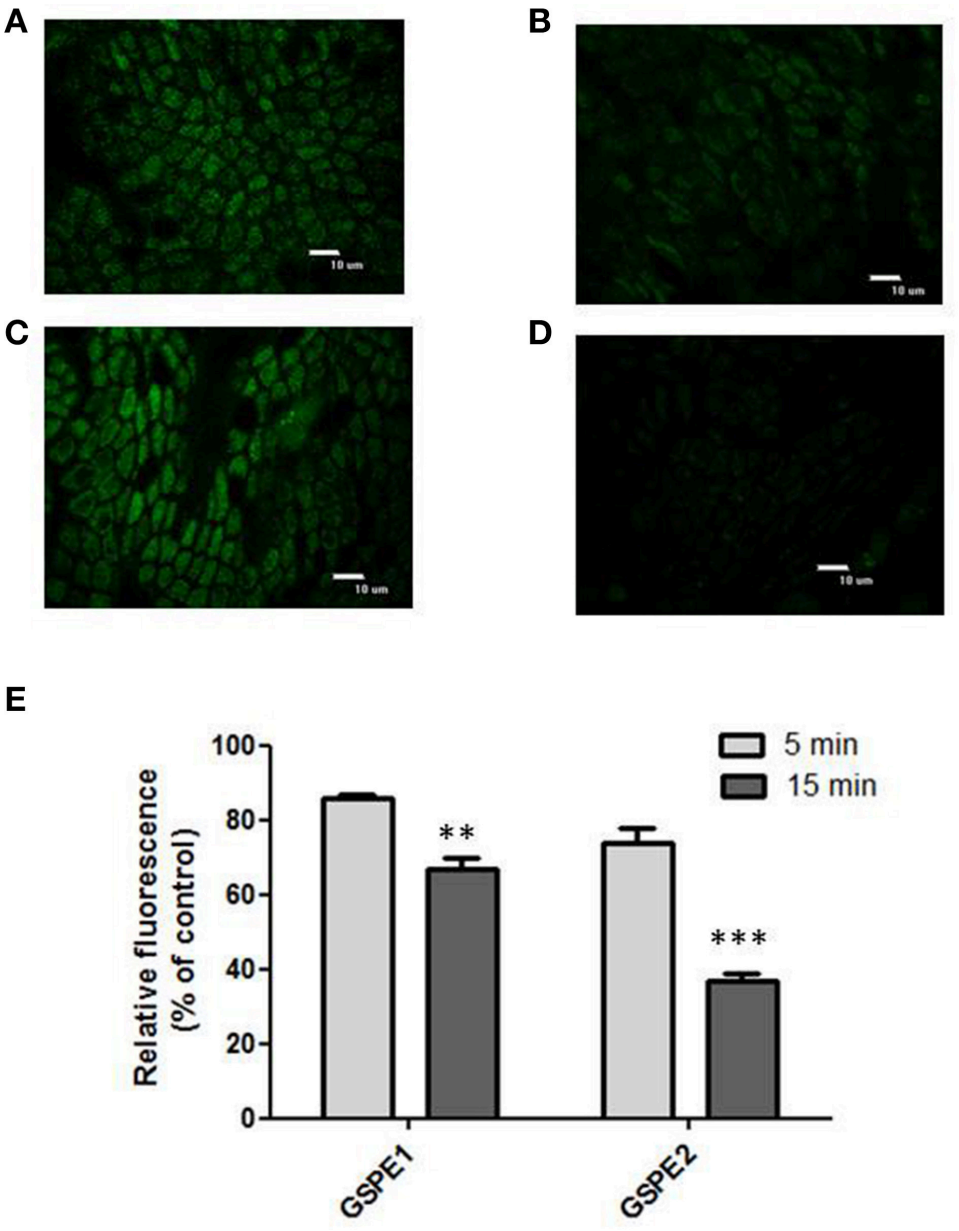
**(A–D)** Effect of polyphenolic extracts incubation on the H_2_O_2_ induced fluorescence. Representative images of CM-H2DCFDA charged superficial colonocytes exposed to 400 μM H_2_O_2_ for 15 min **(A,C)**; representative images of CM-H2DCFDA charged superficial colonocytes pre-incubated with GSPE 1 **(B)** and GSPE 2 **(D)**, respectively for 1 h and then exposed to 400 μM H_2_O_2_ for 15 min. The epithelial surface was visualized using confocal laser scanning microscopy as described in the Materials and Methods Section at 100X objective. **(E)** The graph shows the residual fluorescence (percentage vs. control) recorded from superficial colonocytes of explants pre-incubated with GSPE1 or GSPE2 for 1 h, charged with CM-H2DCFDA and then treated with 400 μM H_2_O_2_ for 15 min. The “control” samples are represented by explants treated with 400 μM H_2_O_2_ for 5 and 15 min, respectively, but not pre-incubated with the extracts. Data are reported as the mean ± SEM of three replicates. The statistical significance of data was assessed by two way ANOVA followed by Bonferroni *post-hoc* test. ^**^P < 0.01; ^***^P < 0.001.

Figures 4A–E shows the effect exerted by the incubation with the polyphenolic extracts on the H_2_O_2_ induced fluorescence in CM-H2DCFDA charged colonocytes. The confocal representative images (Figures 4A–D) show that the preincubation with the extracts was able to decrease the H_2_O_2_ induced fluorescence. The effect was quantified as percentage of residual fluorescence vs. control (control: explants exposed to H_2_O_2_ but not to the extracts) following 5 min and 15 min H_2_O_2_ exposure respectively (Figure 4E). Both extracts were able to significantly inhibit the fluorescence signal at the two times of H_2_O_2_ exposure, but the inhibitory effect was more pronounced following 15 min exposure than 5 min. Moreover, the inhibitory effect exerted by GSP2 appeared significantly increased with respect to the effect exerted by GSP1 as assessed by two ways Anova.

## Discussion

The increase of free radical production and the impairment of antioxidant defenses have been related to the progression of intestinal chronic inflammation diseases (Biasi et al., 2013). Colon is particularly exposed to prooxidant condition (Sanders et al., 2004) and this aspect has been supposed to contribute to the colon greater cancer susceptibility. Maintenance of the correct gastrointestinal redox balance preventing oxidative damage is thus an important aspect for human health. An antioxidant intestinal environment is maintained by a complex dynamic system involving antioxidant enzymes, as well as non-enzymatic molecules some of which originate from the diet (Circu and Aw, 2012). Polyphenols are the most abundant antioxidants in the diet and red grapes represent an excellent source of polyphenolic compounds.

During absorption polyphenols accumulate in the intestinal mucosa, where they reach higher concentrations than in other tissues, and can exert their activities (Biasi et al., 2014) due to both direct antioxidant effects and indirect activation of redox-sensitive cell pathways involved in negative regulation of inflammation and immune modulation.

The present work was addressed to study the intracellular antioxidant activity of red grape polyphenolic extracts in rat superficial colonocytes from freshly isolated explants. Two polyphenol extracts obtained from grapes at different maturation stage (named GSPE1 and GSPE2) were utilized, in order to assess if changes in the polyphenolic composition, due to the ripening process of fruit tissues, can influence the potential intracellular antioxidant activity.

The study was carried out by *in situ* confocal microscopy assessment of intracellular antioxidant activity based on CM-H2DCFDA charged colon explants. A number of methods are available in literature for the measurement of the antioxidant capacity of different antioxidant compounds, but they all use cell free systems and they do not provide any information about the ability of an antioxidant molecule to pass through the plasma membrane and exert its activity within the cells. In order to get insight about the intracellular effects of polyphenols on the functioning epithelial mucosa of the colon, the experimental model chosen in the present work was represented by freshly isolated colon explants, which closely resemble the functional and morphological characteristics of the epithelium *in vivo* (Bjorkman et al., 1986).

H_2_O_2_ was used as oxidative stress inducer since it is a very important oxidative stress agent in colonic epithelial cells for the pathogenesis of inflammatory bowel disease (Uchiyama et al., 2011). Indeed, in chronic colon inflammation, production of H_2_O_2_ by numerous stimulated phagocytes and from some bacteria species present in the gastrointestinal tract is markedly increased (Nathan, 2002; Strus et al., 2009). The H_2_O_2_ concentrations used in this work is consistent with the H_2_O_2_ concentration that can be released by phagocytes and bacteria in chronic inflammatory conditions of the colon. As demonstrated in *in vitro* studies, macrophages and neutrophils are able to produce about 1 mM H_2_O_2_ in 2 h (Becker et al., 2002) and some bacteria species of *Streptococcus, Enterococcus* and *Lactobacillus* genera, present on the mucus membranes, are also able to produce comparable amounts of H_2_O_2_ in aerobic conditions (Ocaña et al., 1999). The use of H_2_O_2_ as oxidative stress inducer is also justified by the finding that the diffusion of H_2_O_2_ through the plasma membrane of mammalian cells is mediated by aquaporin, a class of membrane-spanning proteins that facilitate the diffusion of water and other substrates across the plasma membrane (Miller et al., 2010). In CM-H2DCFDA charged superficial colonocytes the H_2_O_2_ induced fluorescence was time-dependent. In particular, the H_2_O_2_ induced intracellular fluorescence was evident already at 5 min exposure, remained constant in the following 5 min and showed a significant (*p* < 0.001) increase at 15 min exposure with respect to 5 or 10 min. Kalyanaraman et al. (2012) recently outlined that DCF fluorescence cannot be used as a direct measure of intracellular H_2_O_2_ because (1) DCFH does not directly interact with H_2_O_2_ to form the fluorescent DCF, and (2) DCFH can be oxidized to DCF by a number of oxidizing species such as hydroxyl radicals (·OH), compounds I and II generated from the interaction between H_2_O_2_ and peroxidase or heme, NO_2_ arising from the myeloperoxidase/H_2_O_2_/NO_2_−system, hypochlorous acid (HOCl), and reactive species arising from peroxynitrite (ONOO-/ONOOH) decomposition. In the light of these considerations, the particular temporal behavior of the H_2_O_2_ induced fluorescence could not directly reflect a time-dependent increase in the H_2_O_2_ intracellular concentration, but is could be rather the expression of a time-dependent increase in the H_2_O_2_ induced intracellular oxidative conditions. In this view 15 min can represent a temporal threshold in our experimental model for the activation of peroxidative reactions following H_2_O_2_ intracellular permeation.

The CM-H2DCFDA charged colon explants observed under confocal microscopy showed the ability to detect intracellular ROS scavenging activity in superficial colonocytes by intracellularly absorbed antioxidant compounds. In fact a dose-response inhibition of the fluorescence of the intracellular probe was observed following pre-incubation for 1 h with the membrane-permeable antioxidant Trolox, known to be intracellular ROS scavenger (Hamad et al., 2010).

When the explants were incubated with the polyphenolic extracts for 1 h a significant decrease of the H_2_O_2_ induced fluorescence of the cells was observed (Figures 4A–E). In particular the inhibitory effect of the two extracts was quantified following 5 or 15 min exposure to H_2_O_2_. The inhibitory effect exerted by GSPE1 and GSPE2 on the H_2_O_2_ induced fluorescence demonstrates the ability of polyphenols contained in red grapes to cross the plasma membrane and exert a direct intracellular antioxidant activity in the colon surface cells. The stronger effect observed at 15 min H_2_O_2_ exposure with respect to 5 min H_2_O_2_ exposure suggests that the effect of the extract could mainly be exerted on the H_2_O_2_ induced peroxidative reactions, which appear later during the exposure (following 15 min).

The two utilized extracts, corresponding to different maturation stages of the grapes, showed different polyphenolic composition (Table 1). GSPE2 was characterized by a higher content of anthocyanins and a more balanced content in stilbenes with an increased concentration of *trans*-resveratrol with respect to GSPE1. As recently outlined by Colon and Nerín (2016), polyphenols in mixture are able to interact among them producing additive, synergistic or antagonist effects which are difficult to be predicted from the known activities of single compounds. Therefore, differences in the polyphenolic composition and possible differences in the polyphenolic interactions between the two extracts were overall expressed by a different *in vitro* antioxidant capacity, measured as ORAC value, which resulted slightly but significantly increased in GSPE2 with respect GSPE1. The different antioxidant capacity of the two extracts observed *in vitro* (see the ORAC value) is reflected in the different inhibition of the intracellular H_2_O_2_ induced CM-H2DCFDA fluorescence, with GSPE2 more effective (about 20% percentage variation) with respect to GSPE1. It is possible to argue that the different polyphenol composition of the two extract activities can account for the different observed intracellular activity, giving a more effective protection against the H_2_O_2_ induced prooxidant condition.

Therefore, obtained data do not provide evidence of the intracellular H_2_O_2_ concentration reduction operated by the polyphenolic extracts. Rather, they provide information of the general intracellular antioxidant activity and, in turn, protective effect of red grape polyphenolic extracts in superficial colonocytes directly exposed to oxidative stress induced by H_2_O_2_ exposure at a physiological concentration.

In conclusion, obtained results demonstrated in rat colon explants that red grape polyphenolic extracts are able to exert an intracellular antioxidant activity on superficial colonocytes, inducing a protection action against pro-oxidant condition. Extracts obtained from mature grapes showed an increased protective activity. In the current panorama of research on the biological activities of grape as prospective sources of valuable nutraceuticals, these results give a contribution to the knowledge of the effects of grape polyphenols on colon epithelium which is particularly exposed to pro-oxidant condition in both physiological and pathophysiological conditions and prone to cancer susceptibility.

## Author contributions

Conceived and designed the experiments: MG, ML. Performed the experiments: MG, II, RC, GG. Analyzed the data: ML, MG, GG, TS. Wrote the paper: ML, GG, MG.

### Conflict of interest statement

The authors declare that the research was conducted in the absence of any commercial or financial relationships that could be construed as a potential conflict of interest.
